# Management of undernutrition during preconception and pregnancy in an urban setting in North India

**DOI:** 10.3389/fpubh.2024.1405247

**Published:** 2024-08-29

**Authors:** Neeta Dhabhai, Ranadip Chowdhury, Sunita Taneja, Medha Shekhar, Jasmine Kaur, Pratima Mittal, Rupali Dewan, Nita Bhandari

**Affiliations:** ^1^Society for Applied Studies (SAS), New Delhi, India; ^2^Vardhman Mahavir Medical College (VMMC) & Safdarjung Hospital, New Delhi, India

**Keywords:** undernutrition, pregnancy, preconception, BMI, inadequate weight gain

## Abstract

**Introduction:**

The prevalence of underweight in women of reproductive age (WRA) in South Asia remains unacceptably high. Underweight women suffer from lowered immunity, infertility, and a risk of developing non-communicable diseases. In pregnancy, undernutrition results in poor neonatal and maternal outcomes. We present the findings and the management strategy of undernutrition in the preconception and pregnancy phase intervention group in the WING study in low- to lower-middle-income neighborhoods of North India.

**Methods:**

We analyzed data from the Women and Infants Integrated Interventions for Growth Study (WINGS) intervention group. In this individually randomized factorial design trial, 13,500 women were enrolled from low to middle-income neighborhoods of Delhi: 6,722 women in the preconception group and 2,640 from the pregnancy group. Food supplements in the form of locally prepared snacks were given to provide necessary calories and protein requirements as per the Body mass index (BMI) during the preconception period and each trimester of pregnancy. The snacks (sweet or savory) and milk or egg as a source of high-quality protein were delivered at home, and intakes were observed. Individual tracking and close monthly monitoring were done for compliance, besides screening and treatment of infections.

**Results:**

The enrolled women's mean (SD) age was 24.2 (3.1) years. Approximately 35% of women had a height of < 150 cm, and 50% had schooling >12 years. 17% of women in preconception and 14 % in pregnancy intervention groups were Underweight. Approximately two-thirds of underweight women improved 9–12 months after management in the preconception group, and the same proportion improved 4 weeks after management during pregnancy. The proportion of women with inadequate weight gain (IWG) during pregnancy was higher in women who were underweight during preconception.

**Discussion:**

A comprehensive approach to managing undernutrition with high-quality energy-dense food supplementation substantially improved weight gain in women during preconception and pregnancy.

**Clinical trial registration:**

http://ctri.nic.in/Clinicaltrials/pmaindet2.php?trialid=19339%26EncHid=%26userName=societyforappliedstudies, identifier: Clinical Trial Registry India #CTRI/2017/06/008908.

## Introduction

The world's sociocultural, environmental, and economic conditions significantly impact a woman's health, and her biology presents unique challenges. This is particularly evident in low- to middle-income countries (LMICs), where issues of food security, along with social and cultural norms, often restrict a woman's ability to improve her diet (1). Being underweight among women of reproductive age continues to be a significant public health concern; a recent study reported the prevalence of underweight women in LMICs at 15%, with a particularly high prevalence of 28% in South Asia, contributing to an economic burden of 2.5–3.8% of the country's GDP (2–4). While the global prevalence of underweight women decreased by 2%, from 12% in 2000 to 10% in 2016, and most regions saw a decline, South Asia experienced the most substantial decrease (from 27% to 22%) (5).

The prevalence of undernutrition among women of reproductive age in India has declined by 45% over the past decade, from 35.5% in 2005–2006 to 18.7% in 2019–2020). However, according to the National Family Health Survey 2019–2021(NFHS 5), 1 in 5 women are still underweight (6). In neighboring states like Haryana and Uttar Pradesh, the figures remain similar, with Haryana reporting 11.4%−17% and Uttar Pradesh 14%−21%.

Underweight women are a particularly vulnerable group, facing numerous health challenges, including anemia, micronutrient deficiencies, and lowered immunity, which increases their susceptibility to infections such as tuberculosis and reproductive tract infections, as well as infertility and a heightened risk of non-communicable diseases (5). Maternal undernutrition has far-reaching consequences, perpetuating an intergenerational cycle of undernutrition through epigenetic changes and leading to poor obstetric outcomes such as low birth weight (LBW), preterm birth, small for gestational age (SGA) infants, stunting, developmental delays, and increased maternal morbidity and mortality (7).

Barker's hypothesis posits that intrauterine fetal nutrition during pregnancy influences the risk of developing adult diseases and disorders by inducing changes in the DNA. The Developmental Origins of Health and Disease (DOHAD) framework identifies the period around conception as critical in mediating parental influences on the next generation's health, particularly by affecting gamete health and embryogenesis before pregnancy is even diagnosed (8–10). While it is acknowledged that being underweight during pregnancy is a serious issue that requires attention, the preconception period is equally crucial. This phase offers a significant window of opportunity to address health and nutrition deficits before pregnancy, highlighting the need for a pragmatic, life-course approach that provides a continuum of care from preconception through pregnancy.

Based on the above principles, we designed and implemented a comprehensive community-based nutritional intervention as part of the Women and Infants Integrated Interventions for Growth Study (WINGS). This intervention was delivered at home, aiming to provide the recommended caloric and protein intake during the preconception period and the additional caloric needs required during the second and third trimesters of pregnancy (11). While clinical settings often rely on qualitative descriptions and quantitative measures of undernutrition, body size is more commonly used in community settings due to its ease of measurement and effectiveness as a screening tool for nutritional status and health. Therefore, BMI was used to indicate undernutrition (12). Pregnant women with inadequate weight gain during pregnancy were identified and managed according to Institute of Medicine (IOM) guidelines (13, 14).

Currently, there is no comprehensive preconception nutrition intervention program for women of reproductive age in India, except for the weekly prophylactic iron and folic acid supplementation and biannual deworming for anemia (15). For pregnant women, the Integrated Child Development Services (ICDS) provide supplementary nutrition through micronutrient-fortified food, energy-dense food, or take-home rations, which offer approximately 600 calories and 15–20 gm of protein (16). However, this food supplementation is not individualized according to trimester-specific requirements or BMI.

The objective of this study is to present the management strategy for addressing undernutrition during preconception and pregnancy in the intervention groups of the WING study, as well as to report the outcomes and improvements achieved through this approach.

## Methods

### Study design and participants

Data were analyzed from the preconception and pregnancy phase intervention groups of the WING study. We are not presenting the RCT results in this manuscript.

To provide a brief overview, the WING study involved the recruitment of 13,500 women aged 18–30 years who met the eligibility criteria (married, with no or one child, and desiring a second child; exclusions included plans to move out of the study area or delivery outside of Delhi). These women were identified and recruited through a door-to-door survey conducted in Delhi's lower and lower-middle socioeconomic neighborhoods. Upon providing written consent, the women were enrolled and underwent the first randomization to either receive a package of integrated preconception interventions or routine care. They were followed until pregnancy was confirmed or up to 18 months post-enrollment. After pregnancy confirmation via ultrasonography, a second randomization was performed (following additional written consent), assigning pregnant women to either enhanced pregnancy and early childhood care interventions or routine care, continuing until the child reached two years of age. The study commenced on July 17, 2017, and concluded in August 2021. The study's setting, methods and initial results have been published previously (11).

For this study, “Improved” during the preconception phase was defined as a shift to the next higher or normal BMI category (e.g., from < 16 kg/m^2^ to 16–18.49 kg/m^2^ or from 16–18.49 kg/m^2^ to ≥ 18.5 kg/m^2^). Adequate weight gain during pregnancy, starting from the second and third trimesters, was defined according to BMI categories as follows: 0.44–0.58 kg/w for underweight women, 0.35–0.50 kg/week for normal-weight women, 0.23–0.33 kg/week for overweight women, and 0.17–0.27 kg/week for obese women (14).

### Food supplements

Energy-dense snacks were prepared using locally sourced, high-quality raw ingredients (such as cereal, pulses, soya, oil, sugar, salt, milk powder, and peanut butter). Study nutritionists carefully designed the recipes to provide the specific calorie needs required to address undernutrition during the preconception and pregnancy phases. The snacks were culturally acceptable and ready to eat, including savory biscuits and cookies (mathis, namak para), puffed soya granules, and a dry sweet mixture (panjeeri). These snacks were pretested in the community and attractively labeled as “diet for women” or “diet for pregnant women.”

In addition to the snacks, fresh eggs were procured daily, boiled, and provided to the participants. Milk (180 ml) was commercially available, prepacked, and treated at an ultra-high temperature (UHT), with a shelf life of 90 days. Hot-cooked meals were also prepared using broken wheat (dalia), sprouted lentils, and gram flour, with variations in the menu each day. Extra snack packets were made available to the women if they reported sharing the snacks with others.

### Delivery and monitoring

All supplements were delivered to the participants' homes. Locally residing community workers delivered eggs, milk, and hot-cooked meals 6 days a week to the participants through neighborhood depots. The consumption of these supplements was closely observed and documented. If a participant was unavailable during the delivery, repeat visits were made to ensure she received her supplements.

Each participant was assigned a trained accredited social health activist (ASHA)-like worker, referred to as a “Sangini.” These Sanginis made home visits to counsel the women on the study's nutritional interventions, observe supplement intake when possible, deliver and replenish the weekly supply of snacks, and monitor weight gain. They also conducted compliance checks and organized referrals to hospitals or outreach clinic as needed.

The weight of each participant was recorded and updated in real time using an electronic tracker for individual monitoring. To ensure accuracy, weighing machines were calibrated monthly.

### Management of underweight during preconception

The nutrition intervention was designed to supplement the participants' regular diet, aiming to improve their general health and ensure that they entered pregnancy in a nutritionally replete state. To address the protein energy deficit, additional food was provided in the form of energy-dense snacks (either sweet or savory), along with milk or egg as sources of high-quality protein.

Anthropometric measurements (height and weight) were taken at home during enrollment using standardized equipment. Height was measured with a Seca 213 stadiometer, and clothed weight was recorded using a Salter 9509 weighing scale. These measurements were repeated every 3 months until pregnancy was confirmed or for up to 18 months if pregnancy did not occur. Women were classified based on their BMI into normal weight (BMI 18.5–24.99 kg/m^2^), moderately underweight (BMI 16–18.49 kg/m^2^), and severely underweight (BMI < 16 kg/m^2^) (17).

Weight gain of < 500 g/month during the preconception period was defined as inadequate weight gain (IWG). For underweight women, monthly weight measurements were continued until improvement was observed.

For women with a BMI of < 21 kg/m^2^, one egg or 180 ml of milk, providing 70 kcal and 6 g protein, was given to all women 6 days a week to improve diet quality. We chose a cut-off BMI of < 21 kg/m^2^ as it represented the median BMI in this community.

Additionally, women with moderate undernutrition received snacks providing 500 kcal and 6–8 g of protein 7 days a week, while those with severe undernutrition received double the quantity −1,000 kcal with 12–15 g of protein. Women with severe undernutrition were also referred to the study clinic for a detailed medical and nutritional assessment. Food supplements were continued until a normal BMI was achieved, and the modified was adjusted based on changes in the BMI category.

Multiple micronutrient (MMN) tablets, providing half to three-fourths of the recommended dietary allowance (RDA) of various micronutrients, were administered three times a week. Iron folic acid (IFA) tablets were given once weekly for anemia prophylaxis (containing 100 mg elemental iron, 1,500 mcg folic acid, and 15 mcg Vit B12). For mild/moderate anemia (Hb 8 ≤ 12 gm/dl), daily Autrin was given for 3 months. Women with severe anemia (Hb < 8 gm/dl) were referred to a collaborating hospital for investigation and treatment.

Blood samples were collected, and a detailed health questionnaire was administered to screen for anemia and infections. Women with inadequate weight gain (IWG) were referred to the outreach study clinic for infection screening (e.g., RTI/STI, tuberculosis, urinary infections, and dental infections) and management of other medical issues (seasonal fever, chronic diseases, and gastrointestinal ailments). They also received nutritionist advice and counseling. Medical referrals were facilitated to the collaborative hospital for further management. A psychosocial assessment was conducted to identify barriers to compliance, such as depression or other interpersonal and family issues.

### Management of undernutrition in pregnant women

Assuming an average weight gain of 12 kg during pregnancy, the estimated additional calorie requirements were 280 kcal plus 8 g of protein in the second trimester and 470 kcal plus 27 g of protein in the third trimester. Nutrient-dense snacks and milk were provided as sources of high-quality protein to meet these requirements (18). A comprehensive antenatal care (ANC) approach was implemented, ensuring a minimum of eight ANC checks, necessary investigations, and registration at a collaborative hospital. Psychosocial assessment was conducted using the Patient Health Questionnaire 9 (PHQ-9).

Women received daily supplementation of multiple micronutrient supplements (MMS, ~1RDA) throughout pregnancy. Starting from the second trimester, they were also provided with twice-daily calcium (500 mg) plus Vitamin D tablets and iron-folic acid (IFA) based on their hemoglobin status. Weight measurements were taken at pregnancy confirmation (in the first trimester) and were repeated monthly until 32 weeks, biweekly until 36 weeks, and weekly until delivery.

All women with a BMI of < 25 kg/m^2^ received daily food supplements in the form of snacks, provided 7 days a week, and milk (70 kcal plus 6 g protein) 6 days a week throughout pregnancy. Additionally, women with a BMI of < 18.5 kg/m^2^ were given a hot cooked meal, providing 500 kcal plus 20 g protein as their first meal of the day, 6 days a week, until delivery. Inadequate weight gain during pregnancy was defined using the following cut-offs: < 0.44 kg/week for underweight women (BMI < 18.5 kg/m^2^), < 0.35 kg/week for normal-weight women (BMI ≥ 18.5 to 24.99 kg/m^2^), < 0.23 kg/week for overweight women (BMI 25 to 29.99 kg/m^2^), and < 0.17 kg/week for obese women (BMI > 30 kg/m^2^) (12). Women identified as having inadequate weight gain (IWG) were also given an additional hot cooked meal (500 kcal plus 20 g of protein) six days a week until delivery. Study workers conducted twice-weekly home visits to ensure compliance and monitor recovery. Referrals to study clinics were made as needed. Nutritional counseling and focused group discussions with the women and their families were conducted to address dietary challenges and promote behavior change communication.

### Statistical analysis

Sociodemographic characteristics were reported as mean (SD) or proportions, as appropriate. We calculated the proportion of underweight women who improved, as well as the mean (SD) and median (IQR) weight change every 3 months during the preconception period. We also calculated the proportion of inadequate gestational weight gain in each trimester and the proportion of women who responded to management after 4 weeks. During preconception, “Improved” was defined as a shift to the next higher or normal category of BMI: from < 16 kg/m^2^ to 16 to kg/m^2^, and from 16–18.49 kg/m^2^ to ≥18.5 kg/m^2^. Adequate weight gain during pregnancy, starting from the second and third trimesters, was defined as 0.44–0.58 kg/w for underweight women, 0.35–0.50 kg/w for normal-weight women, 0.23–0.33 kg/w for overweight women, and 0.17–0.27 kg/w for obese women (14).

## Results

The sociodemographic characteristics of the 6,722 women of reproductive age (WRA) and 2,460 pregnant women are depicted in Table 1. As per the study design, the cohort of pregnant women was randomized equally into preconception intervention and control groups. The average age was 24 years. About a third of the study population (34.8%) was short stature (< 150 cm). Half of the women had more than 12 years of schooling. The majority (~95%) were homemakers, and more than half lived in joint families. Approximately 17% of women of reproductive age (WRA) and 14% of pregnant women were underweight.

**Table 1:** Baseline characteristics of women of reproductive age at enrollment and pregnant women at second randomization.

**Characteristics**	**WRA (*n* = 6,722)**	**Pregnancy (*n* = 2,460)**
Age, years; mean (SD)	24.2 (3.1)	24.5 (3.1)
Height, cm; mean (SD)	152.2 (5.7)	152.4 (5.7)
Height < 150 cm	2,340 (34.8)	846 (34.4)
**BMI category (kg/m** ^2)^		
≥25 (overweight and obese)	1,798 (26.8)	595 (24.2)
18.5 to 24.99 (normal)	3,774 (56.1)	1,498 (60.9)
16 to 18.49 (moderately underweight)	1,004 (14.9)	328 (13.33)
< 16 (severely underweight)	146 (2.2)	39 (1.5)
Hindu	5,540 (82.4)	2,028 (82.4)
No Schooling	336 (5.0)	110 (4.5)
Joint or extended family^*^	3,922 (58.6)	1,636 (66.5)
Schooling of ≥12 yr.	3,286 (49.1)	1,250 (50.8)
Homemaker	6,328 (94.6)	2,342 (95.2)
Family with below poverty line card	129 (1.9)	84 (3.4)
Family covered by health scheme/health insurance	555 (8.3)	(10.6)

^*^Adult relatives other than the enrolled woman's husband and children living together in a household.

In this study, a joint or extended family was defined as a household where adult relatives other than the enrolled woman's husband and children lived together.

**Figure 1:**
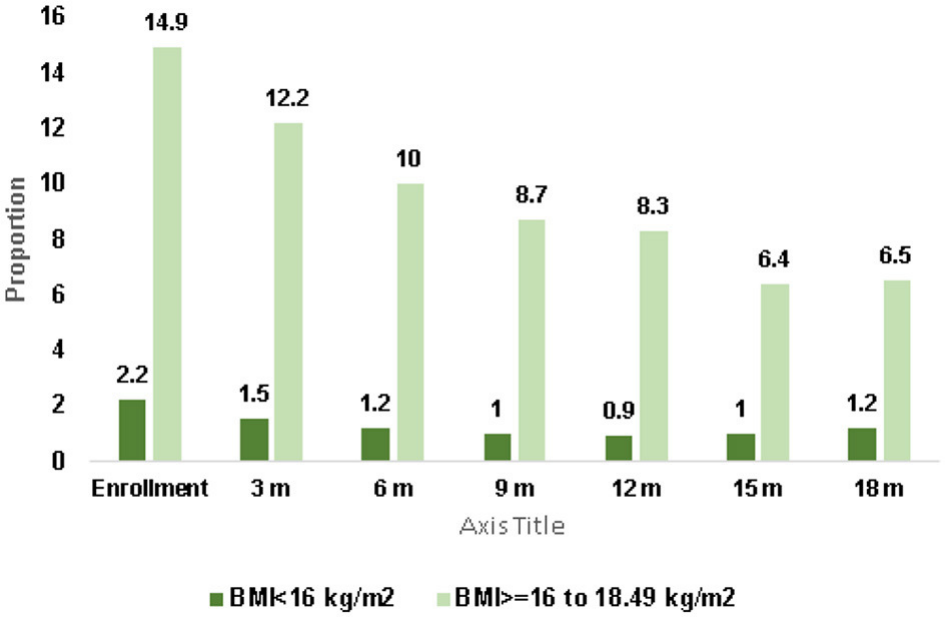
Trend in prevalence of undernutrition in women of reproductive age over 12 months *N* = 6,722.

Figure 1 illustrates the prevalence of undernutrition among women of reproductive age (WRA) for one year following enrollment. At the time of enrollment, 17% of women were underweight, with 2.2% classified as severely underweight (BMI of < 16 kg/m^2^) and 14.9% as moderately underweight (BMI 16–18 kg/m^2^). After 12 months, the proportion of severely underweight women decreased to 0.9%, and more than half of the moderately underweight women showed improvement.

The women with a BMI of ≥18.5 kg/m^2^ at enrolment gained on an average 900 g to 1.5 kg during the follow-up period.

Table 2 shows the response to the management of underweight WRA. Among the severely underweight women, 46% showed improvement after three months, with a mean (SD) weight gain of 1.7 kg (2.4), moving to a higher BMI category (BMI 16–18.49 kg/m^2^). By the end of 9–12 months, 68% of these women showed improvement, with a mean (SD) weight gain of 3.5 kg (3.9). For moderately underweight women, 33% showed improvement after three months, with a mean (SD) weight gain of 1.3 kg (1.7). By 9–12 months, 53% had improved, with a mean (SD) weight gain of 2.8 kg (2.9).

**Table 2:** Response to management of undernutrition in women of reproductive age.

**Severe undernutrition (BMI < 16 kg/m) at enrolment (*n =* 47)**	**Enrollment to 3 m**	**3 to 6 m**	**6 to 9 m**	**9 to 12 m**	**12 to 15 m**	**15 to 18 m**
Moved to a higher category (BMI 16 to 18.49 kg/m^2^)	46.8%	53.2%	57.5%	68.1%	59.6%	59.1%
**Change in weight (kg)**						
Mean (SD)	1.7 (2.4)	2.6 (2.9)	3 (3.3)	3.5 (3.9)	3.6 (4.3)	4.2 (5.4)
Median (IQR)	1.3 (0.4 to 2.5)	1.8 (0.8 to 4.1)	2.2 (0.6 to 5.5)	2.2 (1.4 to 5.5)	3.0 (1.1 to 6.6)	3.4 (0.3 to 7.4)
**Moderate undernutrition (BMI 16 to 18.49 kg/m) at enrolment (*****n** =* **332)**	**Enrollment to 3 m**	**3 to 6 m**	**6 to 9 m**	**9 to 12 m**	**12 to 15 m**	**15 to 18 m**
Moved to higher category (BMI > 18.49 kg/m^2^)	33.1%	41.9%	49.1%	53.3%	59.3%	61.6%
**Change in weight (kg)**						
Mean (SD)	1.3 (1.7)	1.9 (2.1)	2.5 (2.7)	2.8 (2.9)	3.4 (3.3)	3.6 (3.4)
Median (IQR)	1.0 (0.1 to 2.6)	1.8 (0.4 to 3.4)	2.3 (0.7 to 4.0)	2.4 (1 to 4.4)	2.9 (1.1 to 5.2)	3.3 (1.4 to 5.3)

Table 3 shows the proportion of pregnant women with IWG, assessed according to the IOM criteria.

**Table 3:** Inadequate weight gain in pregnancy.

**Period of gestation (weeks)**	**All**	**Baseline BMI**		
		<**18.5 kg/m**	**18.5–24.9 kg/m**	≥**25 kg/m**
		***n** =* **290**	***n** =* **1,125**	***n** =* **457**
Early pregnancy (16–20 weeks (*n =* 1,872)	229.3%	31.7%	31.1%	23.2%
		*n =* 306	*n =* 1,159	*n =* 466
Early pregnancy (20–24 weeks (*n =* 1,931)	23.7%	28.8%	24.5%	18.2%
		*n =* 309	*n =* 1,181	*n =* 481
Mid pregnancy (>24–28 weeks) (*n =* 1,971)	27.8%	35.9%	29.6%	18.1%
		*n =* 286	*n =* 1,192	*n =* 473
Late pregnancy (> 28–32 weeks) (*n =* 1,951)	32.7%	46.5%	35.2%	18.2%

The data reveal that the proportion of women with IWG increased as pregnancy progressed but was prevalent in women with pre-existing undernutrition. Among women with a prepregnancy BMI of < 18.5 kg/m^2^, 29% had IWG at 24 weeks, 36% at 28 weeks, and 47% at 32 weeks of gestation. In comparison, women with a normal prepregnancy BMI (18.5–24.9 kg/m^2^) had IWG rates of 25% at 24 weeks, 30% at 28 weeks, and 35% at 32 weeks of gestation. Overall, the proportion of women with IWG reached 33% at 32 weeks of gestation. The average weight gain from the confirmation of pregnancy until 35–36 weeks was 8.8 kg.

Table 4 shows the response to the management of IWG during pregnancy. It shows that 68% of women with IWG at 20–24 weeks of gestation achieved adequate weight gain at 24–28 weeks. Similarly, 62% of pregnant women with IWG at 28–32 weeks and 64% women at 28–32 weeks showed gestational weight gain in the following 4 weeks.

**Table 4:** Response to management of inadequate weight gain in pregnancy.

**Period of gestation (weeks)**	**Women with inadequate weight gain *n* (%)**	**Adequate weight gain in the next 4 weeks**
Early pregnancy (16 to 20 weeks), *n =* 1,872	548 (29.3)	73.9%
Early pregnancy (20 to 24 weeks), *n =* 1,931	457 (23.7)	68.1%
Mid pregnancy (>24 to 28 weeks), *n =* 1,971	547 (27.8)	61.5%
Late pregnancy (>28–32 weeks), *n =* 1,951	638 (32.7)	63.9%

## Discussion

In this study, of the 6,722 women in the preconception group, 1,150 women (17%) were underweight, with 1,004 women being moderately underweight and 146 women being severely underweight. Approximately two-thirds of severely undernourished women and half of those who were mildly to moderately undernourished women showed improvement over 12 months of management during the preconception period.

The prevalence of inadequate weight gain ranged from 24 to 33% during early, mid, and late pregnancy, peaking in late pregnancy. This was notably higher among women with a preconception BMI of < 18.5 kg/m^2^. The data effectively demonstrate that approximately two-thirds of pregnant women with inadequate gestational weight gain achieved adequate weight gain after 4 weeks of management. As pregnancy progresses, the energy intake required to support maternal and fetal metabolism (energy expenditure), fetal growth, and energy storage increase (energy storage) increases (19). Underweight women, therefore, are most vulnerable during the third trimester, where this demand peaks, as evidenced in our study. The continuation of conditions that result in undernutrition during the preconception period impedes adequate gestational weight gain, highlighting the need for early detection and management. The International Federation of Obstetricians and Gynecologists (FIGO) recommends estimating BMI at every opportunity of interaction with non-pregnant women of reproductive age and providing appropriate counseling to identify women at nutritional risk early (20).

Most LMICs do not include preconception screening strategies in their national health guidelines, and the significance of preconception nutrition for women of reproductive age is often overlooked. A desk review of studies conducted over the last decade in South Asian countries to gather evidence on nutritional strategies emphasized the need for early pregnancy registration to screen for severe undernutrition (21).

A recent analysis of the ICDS scheme found only a 57.8% of pregnant and lactating women utilized the service (22). Factors such a taste preferences, inaccessibility, service disruption, and sharing of food supplements were cited barriers to utilization. In this study, we addressed these challenges by tailoring interventions to individual needs and delivering them at home to enhance compliance. A similar (pilot) maternal nutrition project implemented in West Bengal, India, aimed to strengthen the existing government food supplementation program in Anganwadi centers for pregnant mothers by assessing and monitoring BMI, providing home counseling, and spot feeding of cooked food, resulting in improved outcomes (23).

The key strengths of our approach included a comprehensive strategy to address undernutrition at the community level, regular monitoring and screening for IWG, home delivery of pretested, culturally acceptable food supplements, tailored calorie and protein supplementation, observed intake, treatment of infections, and consistent follow-up. Height and weight assessments were standardized and subjected to quality checks.

Implementing this strategy posed several challenges, including ensuring consistent quality at every stage of preparation, maintaining adequate supplies, and ensuring timely delivery. A large team was necessary for these tasks, which could present a programmatic challenge in scaling up the intervention. During the COVID-19 lockdown periods—from 24^th^ March to 18^th^ May 2020 (55 days), and from 1^st^ April to 1^st^ June 2021 (61 days)—the delivery of food supplements to some areas was disrupted, necessitating the discontinuation of hot cooked meals and the provision of precooked snacks instead. This may have affected some outcomes. Additionally, managing costs within the study budget was challenging. We also had to address issues related to women sharing snacks with their families, especially children, and frequently substituting their regular diet with the additional food provided.

An in-depth qualitative analysis of the causes of undernutrition would have provided more nuanced insights, but this was a limitation in our statistical analysis. We acknowledge that while BMI is not the ideal measure of weight status and undernutrition, it remains an invaluable screening tool for community trials (12).

This study has tremendous public health implications. Preconception care, particularly the nutritional assessment of women of reproductive age, represents a major unmet need in India and South Asia. Our findings highlight the absence of preconception nutrition programs, even though India has robust guidelines and programs targeting child undernutrition, including support for pregnant and lactating women. However, the pre-pregnancy phase in women of reproductive age remains largely unaddressed. Poshan Abhiyan, launched in 2018, adopts an integrated approach but lacks a specific preconception component beyond anemia prevention (23). Overall, in the WING trial, the integrated interventions led to a 24% reduction in low birth weights and a 51% reduction in stunting at 24 months of age compared to the control group. The interventions also yielded improved maternal outcomes, including higher hemoglobin concentration, better gestational weight gain, and reduced risks of reproductive tract infections, anemia, and pregnancy-induced hypertension.

### Programmatic implications

Currently, preconception and early pregnancy screening for underweight women is not part of any national program in India. There is a need for education and advocacy on proper nutrition, using IEC material by grassroots workers to promote social and behavioral change. Early pregnancy screening for underweight women and consistent reporting by health workers whenever women of reproductive age are examined could be effective in addressing this issue. Family planning programs could incorporate BMI as a screening tool for undernutrition in women of reproductive age when identifying eligible couples, and subsequently include them in ICDS programs. The existing ICDS program could be adapted to provide trimester-specific food supplements during pregnancy. Conducting an implementation research project to study the feasibility of these interventions would be a positive step forward.

## Conclusion

The promotion of preconception nutrition is critical and requires greater focus, as undernutrition needs to be managed before pregnancy, often requiring 9–12 months for improvement. Our study shows that supplementation with high-quality, energy-dense food, coupled with close monitoring, individual tracking for compliance, screening, and treatment of infections and other morbidities, as well as observed intake, significantly improved the nutritional status of women during preconception and pregnancy.

## Data Availability

The raw data supporting the conclusions of this article will be made available by the authors, without undue reservation.
