# Supplementary material for: Management of undernutrition during preconception and pregnancy in an urban setting in North India
Source: Front Public Health. 2024 Aug 29;12:1405247. doi: 10.3389/fpubh.2024.1405247 (PMC11390395; doi:10.3389/fpubh.2024.1405247)
Supplement: Supplementary file 1 [file Data_Sheet_1.docx]

**Table 1. Summary of the intervention package WINGS**

| **Period** | **Health** | **Nutrition** | **Psychosocial Support** | **WASH** |
| --- | --- | --- | --- | --- |
| Pre- conception | Screen & treat medical conditions | Provide IFA & MMN,  Provide egg/milk if BMI <21, Screen & treat malnutrition, anemia | Promote positive thinking & problem-solving skills | Promote personal, menstrual and hand hygiene |
| Pregnancy | >8 antenatal contacts, screen & treat GHT/pre-eclampsia, GDM, hypothyroidism, UTI, RTI, calcium supplement | Provide IFA & MMN,  Locally-prepared snacks (210/400 kcal, 2/21gm protein in 2nd/3rd trimester),  Provide milk (180 ml daily),  Provide one additional hot cooked meal (500 kcal, 20gm protein) if BMI<18.5 or inadequate gestational weight gain | Promote positive thinking & problem-solving skills | Provide water filters, soap, hand washing station, disinfectant |
| Early childhood | Empower family to identify danger signs and seek care early | 0-6 mo: lactation support for early and exclusive BF  6-24 mo: promote timely CF & continued BF  Provide supplementary food (125/250 kcal, 2.5/5gm protein 6-11/12-23 mo)  Double supplement if inadequate weight gain | Promote early child play and responsive care | Provide play mat and potty |
| Postnatal 6 mo: mother | Facilitate postnatal visit at 6 wk | Provide IFA, MMN, calcium & Vit D,  Locally-prepared snacks (600 kcal, 20gm protein),  provide milk (180 ml daily) | Promote positive thinking & problem-solving skills | Same as in pregnancy |

**Control group:** Weekly iron-folic acid supplementation as part of the national program during preconception period; routine antenatal and postnatal care for the mother and routine newborn and infant care during early childhood

**Figure 1. Study Flow**

Non-pregnant, 18-30 years old married women,

with no or one child who wish to have a child: 20243

6778 (50·2%)

No preconception interventions

6722 (49·8%)

Preconception interventions

6743 excluded

640 Temporary housing

3130 Moving away

2973 Refusals

13500 (66.7%) Underwent first randomization

3594

Pregnancies confirmed

338 Non-consent 266

604 Moving away 563

3098

Pregnancies confirmed

1326 Preconception and Pregnancy and Early childhood interventions

Group A

2652 (73·8%) underwent second randomization

1326 Preconception interventions only

Group B

(Group B)

2269 (73·2%) underwent second randomization

1134 Pregnancy and Early childhood interventions only

Group C

(Group C)

1135 Control group

Group D

(Group D)

**Table 2 Compliance to intervention in Preconception N= 6722**

| **Preventive and Promotive Interventions** | **Enrollment to 6 mo** | **6 to 12 mo** | **12 to 18 mo** |
| --- | --- | --- | --- |
| **Nutrition** |  |  |  |
| Median (IQR) percent weeks ferrous fumarate plus folic acid consumed by women (anemia prophylaxis), n=6668, 4989, 3820 | 88.9 (71.4 to 100) | 86.7 (60 to 100) | 85.7(50 to 100) |
| Median (IQR) percent days multiple micronutrients consumed, n=6638, 4887, 3765 | 66.7 (50 to 80.8) | 70.8 (46.7 to 85.7) | 66.7 (33.3 to 81.0) |
| Median (IQR) percent days egg or milk consumed by women with BMI <21 kg/m2, n=2791, 1547, 939 | 92 (80.8 to 95.5) | 90.6 (79.3 to 95.7) | 93.4 (83.3 to 97.0) |
| Median (IQR) percent days snacks consumed by women with BMI <18.5 kg/m2, n=1224, 563, 343 | 73.2 (50 to 87.2) | 69.4 (50 to 80.4) | 71.4 (49.0 to 83.3) |

**Table 3 Pregnancy compliance to interventions N=2460**

| **Nutrition** |  |
| --- | --- |
| Median (IQR) percent days micronutrient supplement consumed, n=2460 | 81.8 (71.4 to 88.9) |
| Median (IQR) percent days iron-folic acid consumed n=2460 | 83.3 (75.0 to 89.6) |
| Median (IQR) percent days calcium and Vitamin D supplement consumed, n=2460 | 81.7 (72.7 to 88.1) |
| Median (IQR) percent days snacks consumed by women with BMI <25 kg/m^2^, n=1865 | 73.0 (58.3 to 82.9) |
| Median (IQR) percent days egg or milk consumed by women with BMI <30 kg/m^2^, n=2355 | 83.3 (71.4 to 90.5) |
